# Serum Resolvin D1 and Maresin 1 Levels During Migraine Attacks and the Interictal Period: A Paired Analysis in Emergency Department Patients

**DOI:** 10.3390/biomedicines13122980

**Published:** 2025-12-04

**Authors:** Miraç Koç, Özge Özen Gökmuharremoğlu, Neslihan Cihan Çalışgan

**Affiliations:** 1 Department of Emergency Medicine, Faculty of Medicine, Kastamonu University, Kastamonu 37150, Turkey; 2 Department of Neurology, Faculty of Medicine, Kastamonu University, Kastamonu 37150, Turkey; 3 Department of Biochemistry, Gaziantep Cengiz Gökçek Maternity and Child Hospital, Gaziantep 27010, Turkey

**Keywords:** migraine, Resolvin D1, Maresin 1, biomarker, emergency medicine, neuroinflammation, Omega-3 fatty acids

## Abstract

**Background/Objectives**: Neurogenic inflammation plays a key role in migraine pathophysiology. This study aimed to evaluate serum Resolvin D1 (RvD1) and Maresin 1 (MaR1) levels during acute migraine attacks and in the interictal period in the same patients, using a paired design. **Methods**: This prospective case–control study included 55 migraine patients and 53 healthy controls. Serum samples were obtained from each patient at two time points: during an acute attack and during the subsequent interictal period. RvD1 and MaR1 concentrations were measured using ELISA. **Results**: Serum RvD1 and MaR1 levels were significantly lower during migraine attacks compared with both the interictal period (*p* = 0.006 and *p* = 0.001, respectively) and healthy controls (*p* < 0.001). Receiver operating characteristic (ROC) analyses showed moderate discriminatory ability for RvD1 (AUC 0.719) and MaR1 (AUC 0.730). **Conclusions**: Reduced RvD1 and MaR1 levels are associated with the acute migraine state. These findings indicate an association rather than a causal mechanism and suggest that SPM measurements may have potential as supportive adjunct biomarkers, although further validation is required.

## 1. Introduction

Migraine is a highly prevalent neurological disorder and a major cause of disability worldwide. Acute migraine attacks frequently lead to emergency department (ED) visits, where they constitute a substantial proportion of headache-related presentations and pose diagnostic and therapeutic challenges for clinicians [1,2]. Despite advances in the understanding of migraine pathophysiology, the absence of objective biomarkers that reflect the dynamic inflammatory state of an attack remains a significant limitation in acute care settings.

Activation of the trigeminovascular system is a central mechanism in migraine pathophysiology. This activation results in the release of vasoactive neuropeptides—particularly calcitonin gene-related peptide (CGRP)—and subsequent sterile neurogenic inflammation characterized by vasodilation, plasma extravasation, and mast cell activation [3,4]. Given this inflammatory cascade, there is growing interest in identifying biomarkers that may help characterize the biological state of a migraine attack and complement clinical evaluation in the ED [5,6].

Specialized pro-resolving mediators (SPMs) have emerged as critical regulators of the active resolution phase of inflammation. Derived from omega-3 polyunsaturated fatty acids (ω-3 PUFAs), mediators such as Resolvin D1 (RvD1) and Maresin 1 (MaR1) help terminate inflammation and restore tissue homeostasis [7,8]. Experimental models have demonstrated that RvD1 and MaR1 reduce neuroinflammation and inhibit nociceptor activity [9], suggesting that they may be relevant to migraine pathobiology. Although several studies have examined the clinical effects of ω-3 PUFA supplementation—including reductions in migraine frequency and severity—these findings provide only indirect evidence regarding endogenous SPM biology in migraine [10,11,12,13,14,15,16].

To date, studies specifically evaluating circulating RvD1 and MaR1 levels in migraine remain scarce. While a few investigations have examined other inflammatory or lipid mediators during migraine episodes—such as cytokines, magnesium, or non-SPM lipid derivatives [9,17]—to the best of our knowledge, no study has directly measured RvD1 or MaR1 using paired samples obtained during both the acute attack and interictal period. Understanding whether these specialized pro-resolving mediators fluctuate across migraine phases may therefore provide novel insight into the inflammatory–resolutive balance in migraineurs. Accordingly, the primary objective of this study was to measure serum RvD1 and MaR1 concentrations during acute migraine attacks and in the subsequent interictal period and to compare these levels with those of healthy controls. A secondary objective was to evaluate the discriminatory performance of these mediators using receiver operating characteristic (ROC) analysis.

## 2. Materials and Methods

### 2.1. Study Design and Ethical Approval

This prospective, single-center, paired case–control study was conducted at the Emergency Department of Kastamonu Training and Research Hospital between 15 January and 15 March 2024. The study protocol was approved by the Kastamonu University Clinical Research Ethics Committee (Approval No: 2023-KAEK-159) and conducted in accordance with the Declaration of Helsinki. Written informed consent was obtained from all participants. Reporting followed STROBE (Strengthening the Reporting of Observational Studies in Epidemiology) guidelines.

### 2.2. Study Population and Groups

Participants were enrolled into three groups:

1. Migraine Attack Group (n = 55)

Episodic migraine without aura (ICHD-3) presenting ≤2 h from onset with VAS ≥ 7/10.

2. Interictal Migraine Group (n = 55)

The same patients reassessed ≥7 days after complete resolution of symptoms (VAS 0/10), confirmed via headache diary.

3. Healthy Control Group (n = 53)

Age- and BMI-matched volunteers without migraine or systemic disease.

All participants were recruited from the same geographical region (Kastamonu Province) using consecutive sampling to minimize selection bias.

Exclusion criteria included: Use of analgesics/NSAIDs/prophylaxis within 1 week, omega-3 supplementation within 3 months, chronic inflammatory disease, hepatic/renal impairment (AST/ALT > 2× ULN; eGFR < 60 mL/min/1.73 m^2^), bleeding disorders, anticoagulant use, pregnancy/lactation, and trauma/surgery within 30 days.

A 7-day medication-free interval was selected based on existing headache research standards, which commonly use a 5–7-day washout period to minimize the short-term pharmacodynamic effects of analgesics and prevent acute treatment carryover [18]. However, we acknowledge that this duration may not eliminate all medication influences and therefore represents a methodological limitation.

### 2.3. Power Analysis and Sample Size

Because no prior study has quantified circulating RvD1 or MaR1 in migraine, power analysis was based on effect size estimates from Ceylan et al.’s PTX3 migraine data (Cohen’s d ≈ 0.98) [19]. PTX3 was selected as a proxy because both markers are linked to neuroinflammatory pathways, although a direct correlation is unproven.

An a priori G*Power v3.1.9.7 (Heinrich Heine University Düsseldorf, Düsseldorf, Germany; https://www.psychologie.hhu.de/arbeitsgruppen/allgemeine-psychologie-und-arbeitspsychologie/gpower, accessed 15 January 2024) analysis indicated 18 participants per group for 80% power at α = 0.05. To compensate for potential dropouts and to increase paired-comparison robustness, we enrolled 55 patients and 53 controls, exceeding the minimum requirement.

### 2.4. Blood Sample Collection and Processing

Attack samples: Drawn within 1 h of ED arrival, prior to treatment, after 10 min of rest.

Interictal + control samples: Collected fasting (≥8 h) between 08:00 and 10:00 a.m. to reduce circadian variation.

Because fasting and circadian timing could not be standardized during acute attacks, this pre-analytical difference is acknowledged as an important limitation.

Samples were collected in serum separator tubes (VacuSEL, Konya, Türkiye), allowed to clot for 30 min, and centrifuged at 2000× *g* for 10 min at 4 °C using a Nüve NF 3000R centrifuge (Nüve Corporation, Ankara, Türkiye). Serum was aliquoted into sterile Eppendorf tubes (Eppendorf SE, Hamburg, Germany) and temporarily stored at −20 °C for ≤24 h, then transferred to −80 °C for long-term storage. Although all samples underwent identical processing, short-term −20 °C storage may affect lipid mediator stability and is reported as a limitation. The ELISA reading and long-term −80 °C storage were performed externally at Farmasina Laboratory (Istanbul, Türkiye) using validated laboratory equipment.

All samples were analyzed within 3 months and freeze–thaw cycles were not allowed.

### 2.5. Biochemical Analyses

#### 2.5.1. ELISA Methodology

Serum RvD1 and MaR1 were measured using commercial ELISA kits (SunRed Biotechnology, Shanghai, China; RvD1 Cat#201129313; MaR1 Cat#201127339).

To eliminate inter-plate variability, paired (attack + interictal) samples from the same patient were analyzed on the same day and on the same ELISA plate. Optical density was read at 450 nm. Concentrations were calculated using 7-point standard curves (R^2^ > 0.95). Samples with CV > 10% were re-run.

#### 2.5.2. Detection Limits and Quality Control

**Assay characteristics (manufacturer-verified):**


**Measuring Range**

**Sensitivity (LOD)**
RvD10.15–30 ng/mL0.114 ng/mLMaR17.5–2000 pg/mL7.247 pg/mL


Values below the LOD were assigned LOD/√2, a standard imputation method for left-censored biomarker data.

Intra-assay CV < 10%; inter-assay CV < 12%.

Cross-reactivity was <0.5% for RvD1 and <0.3% for MaR1.

### 2.6. Statistical Analysis

Statistical analyses were conducted using SPSS 22.0 (IBM Corp., Armonk, NY, USA) and GraphPad Prism 9.0 (GraphPad Software, San Diego, CA, USA).

- Normality: Shapiro–Wilk
- Continuous variables: mean ± SD or median (IQR)
- Categorical variables: n (%)

Given the paired design,

- Paired comparisons: Paired t-test or Wilcoxon signed-rank test
- Independent comparisons: Independent t-test or Mann–Whitney U test

ROC analysis included AUC, 95% CI, Youden index, sensitivity, and specificity.

Bonferroni correction was applied for three planned pairwise comparisons (attack vs. interictal, attack vs. control, interictal vs. control): adjusted α = 0.05/3 = 0.0167.

A two-tailed *p* < 0.05 (and Bonferroni-corrected *p* < 0.0167) was considered statistically significant.

## 3. Results

### 3.1. Participant Characteristics

A total of 108 participants were included in the final analysis: 55 patients with episodic migraine and 53 healthy controls. Female predominance was observed in both the migraine group (n = 43, 78.2%) and the healthy control group (n = 38, 71.7%), with no statistically significant difference in gender distribution between the groups (*p* = 0.437). The groups were well-matched for age, with an identical median age of 38 years and no significant difference observed (*p* = 0.540). Demographic details are summarized in Table 1.

### 3.2. Serum Levels of Resolvin D1 and Maresin 1

**Figure 1:**
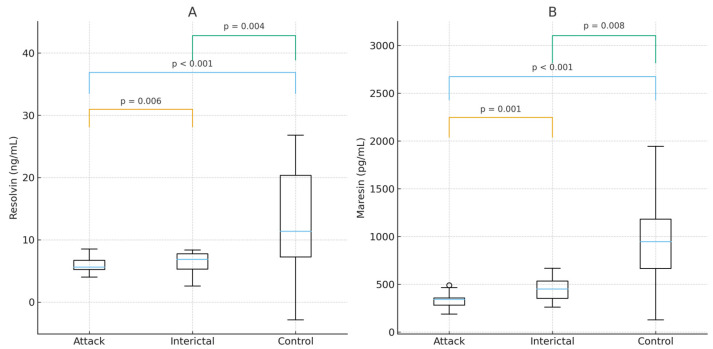
Serum Concentrations of Pro-resolving Mediators (RvD1 and MaR1). Boxplots illustrating serum levels of (**A**) Resolvin D1 (RvD1) and (**B**) Maresin 1 (MaR1) measured in migraine patients during acute attacks, the subsequent interictal phase, and in healthy controls. Boxes represent interquartile ranges (IQR), horizontal lines indicate medians, and whiskers indicate data range (minimum to maximum). Horizontal bars indicate pairwise group comparisons with corresponding *p*-values.

Serum concentrations of both pro-resolving mediators were lowest during the migraine attack phase and significantly reduced in migraine patients compared with healthy controls (Figure 1).

RvD1:

- Attack: 5.84 ng/mL (IQR: 4.59–8.17)
- Interictal: 6.40 ng/mL (IQR: 5.69–10.84)
- Controls: 12.01 ng/mL (IQR: 6.59–26.25)

Paired analysis showed significantly lower RvD1 levels during attacks compared with the interictal phase (*p* = 0.006). Interictal levels were also significantly lower than controls (*p* = 0.004).

All comparisons remained significant after Bonferroni correction (adjusted α = 0.008).

MaR1:

- Attack: 339.20 pg/mL (IQR: 273.26–593.32)
- Interictal: 417.03 pg/mL (IQR: 358.56–745.10)
- Controls: 751.34 pg/mL (IQR: 391.95–1837.40)

Paired comparison showed significantly lower MaR1 levels during attacks than interictal (*p* = 0.001). Interictal levels were also significantly lower than controls (*p* = 0.008).

All comparisons remained significant after Bonferroni adjustment.

Comprehensive statistical comparisons are provided in Table 2.

### 3.3. Diagnostic Performance of RvD1 and MaR1

**Figure 2:**
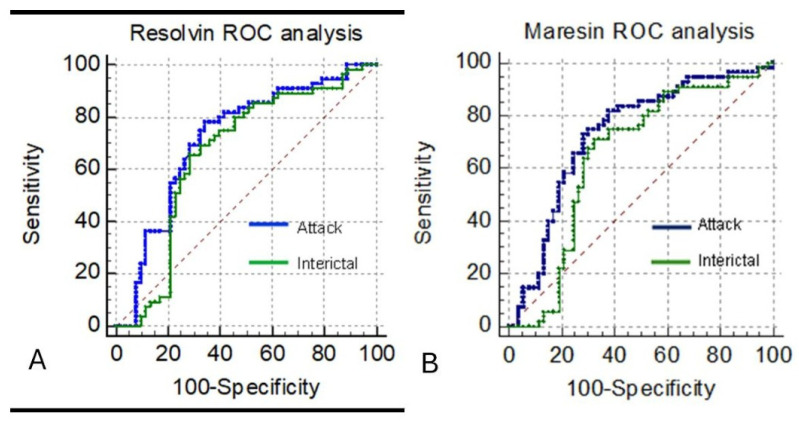
ROC Curves Showing Diagnostic Performance of Serum RvD1 and MaR1. Receiver operating characteristic (ROC) curves demonstrating the ability of serum levels of (**A**) Resolvin D1 (RvD1) and (**B**) Maresin 1 (MaR1) to differentiate migraine patients from healthy controls. The blue curves represent discrimination between the acute attack phase and controls, while the green curves represent discrimination between the interictal phase and controls. The red dashed diagonal line represents the line of no discrimination (reference line).

ROC analyses were performed to evaluate diagnostic discrimination (Figure 2, Table 3).

### 3.4. Acute Attack Phase

- RvD1: AUC = *0.719* (95% CI: 0.624–0.801; *p*< 0.001)
  Cut-off ≤ 8.51 ng/mL → Sensitivity 78.2%**,** Specificity 66.0%, PPV 70.5%, NPV 74.5%
- MaR1: AUC = *0.730* (95% CI: 0.636–0.811; *p*< 0.001)
  Cut-off ≤ 460.02 pg/mL → Sensitivity 72.7%, Specificity 71.7%, PPV 72.7%, NPV 71.7%

### 3.5. Interictal Phase

- RvD1: AUC = 0.662 (95% CI: 0.550–0.750; *p* = 0.004)
  Sensitivity 65.5%, Specificity 71.7%
- MaR1: AUC = 0.648 (95% CI: 0.631–0.737; *p* = 0.009)
  Sensitivity 70.9%, Specificity 67.9%

Both biomarkers demonstrated moderate discriminatory ability, with higher performance during the acute attack phase compared with the interictal period. Because PPV and NPV are prevalence-dependent metrics, their values in this single-center cohort may not directly generalize to other populations. Therefore, likelihood ratios—which are independent of disease prevalence—may provide complementary information for clinical interpretation.

## 4. Discussion

In this study, we found that serum levels of the specialized pro-resolving mediators Resolvin D1 (RvD1) and Maresin 1 (MaR1) were significantly lower in patients with episodic migraine compared with healthy controls, with the lowest concentrations observed during acute attacks [9]. Although the study design precludes causal inference, these findings suggest an association between reduced SPM levels and the migraine state. The persistence of lower RvD1 and MaR1 levels in the interictal period may indicate that biochemical recovery following an attack does not occur immediately; however, whether this reflects residual neuroinflammatory activity or other physiological factors cannot be determined from the available data [20,21].

Our observations show partial similarities to findings reported in other neurological conditions involving acute inflammatory activation. Previous studies have described decreased levels of RvD1 in acute ischemic stroke and traumatic brain injury, and reductions in MaR1 in various inflammatory and neuropathic models [8,22,23,24,25]. Although these data support the concept that SPMs may fluctuate in the context of neuroinflammation, such comparisons should be interpreted cautiously because the underlying mechanisms differ substantially from those of migraine. The present findings contribute to this broader literature by characterizing SPM behavior specifically in the context of migraine attacks and the interictal phase.

From a clinical standpoint, the moderate discriminatory performance of RvD1 and MaR1 in ROC analyses (AUC values 0.66–0.73) suggests that these mediators are unlikely to serve as standalone diagnostic biomarkers. However, their ability to differentiate between attack and non-attack states, particularly during the acute phase, raises the possibility that they may hold adjunctive value in situations where diagnostic uncertainty exists. Further validation studies in larger, multicenter cohorts with standardized sampling procedures are required before any clinical application can be considered.

Dietary and metabolic pathways related to omega-3 polyunsaturated fatty acids—precursors for SPM biosynthesis—have been explored in the context of migraine [26,27]. Meta-analyses have reported that omega-3 supplementation may reduce migraine frequency and severity [12,13,14,15,28]. Although our data do not establish a mechanistic link, the observed reductions in RvD1 and MaR1 during migraine attacks may provide biochemical context compatible with these clinical observations [29,30]. Whether interventions aimed at supporting endogenous resolution pathways can influence migraine burden remains an important question for future interventional research.

Several methodological considerations should also be highlighted. Because no prior studies have quantified RvD1 or MaR1 levels in migraine patients, the power analysis relied on effect size estimates derived from PTX3, a different inflammatory biomarker [19]. This proxy-based approach represents a methodological limitation and may have influenced the precision of sample size estimation. Additionally, SPM concentrations vary according to circadian rhythm and nutritional status [9]. Although fasting morning samples were collected from interictal and control participants, this could not be standardized during acute attacks, potentially introducing pre-analytical variability. Sample storage represents another consideration: serum was temporarily stored at −20 °C for up to 24 h before transfer to −80 °C. Given the sensitivity of lipid mediators, partial degradation during this initial storage period cannot be fully excluded, even though all samples underwent identical handling. These limitations should be taken into account when interpreting the findings.

In summary, our study demonstrates that serum RvD1 and MaR1 levels are significantly lower in patients with migraine compared with healthy individuals, particularly during acute attacks. While these mediators show moderate discriminatory capability, they should not be considered definitive diagnostic tools at this stage. Instead, they may offer insight into the inflammatory–resolutive balance underlying migraine. Further mechanistic, longitudinal, and interventional studies are needed to elucidate the biological significance of these findings and determine whether SPM-focused approaches may hold future clinical relevance.

### Limitations

This study has several limitations. First, because no prior research has evaluated circulating RvD1 or MaR1 levels in migraine patients, the sample size was estimated using effect size data from PTX3, a distinct inflammatory biomarker. This proxy-based approach may have limited the precision of the power calculation. Second, SPM concentrations are influenced by circadian rhythm and nutritional status. Although fasting morning samples were collected from interictal and control participants, such standardization was not feasible during acute attacks, potentially introducing pre-analytical variability. Third, serum samples were initially stored at −20 °C for up to 24 h before being transferred to −80 °C. Given the biochemical sensitivity of lipid mediators, partial degradation during this period cannot be entirely excluded. Finally, this was a single-center study with a moderate sample size, which may restrict generalizability, and only two SPMs were measured rather than a broader lipid mediator panel.

## 5. Conclusions

In conclusion, serum levels of the specialized pro-resolving mediators RvD1 and MaR1 were significantly lower in migraine patients than in healthy controls, with the most pronounced reductions observed during acute attacks. Although these mediators demonstrated only moderate discriminatory performance, they may provide insights into the inflammatory–resolutive balance associated with migraine. Further research, particularly longitudinal and interventional studies, is needed to clarify the biological significance of these findings and to determine whether modulation of resolution pathways may hold clinical relevance in migraine management.

## Figures and Tables

**Table 1:** Demographic Data of Migraine and Control Groups.

Variables	Migraine Group (n = 55)	Control Group (n = 53)	*p*-Value
Age	38 (32–44)	38 (31–45)	0.540
Gender			0.437
Male	12 (21.8%)	15 (28.3%)	
Female	43 (78.2%)	38 (71.7%)	

Data are presented as median (interquartile range, IQR) for continuous variables, and number (percentage) for categorical variables. *p*-values indicate statistical comparisons between groups. IQR, interquartile range.

**Table 2:** Comparison of Serum Resolvin D1 and Maresin 1 Levels Between Study Groups.

Biomarker	Migraine Attack Group (n = 55) Median (IQR)	Migraine Interictal Group (n = 55) Median (IQR)	Control Group (n = 53) Median (IQR)	*p*-Value
RvD1 (ng/mL)	5.84 (4.59–8.17)	—	12.01 (6.59–26.25)	<0.001
RvD1 (Interictal vs. Control)	—	6.40 (5.69–10.84)	12.01 (6.59–26.25)	0.004
RvD1 (Attack vs. Interictal)	5.84 (4.59–8.17)	6.40 (5.69–10.84)	—	0.006
MaR1 (pg/mL)	339.20 (273.26–593.32)	—	751.34 (391.95–1837.40)	<0.001
MaR1 (Interictal vs. Control)	—	417.03 (358.56–745.10)	751.34 (391.95–1837.40)	0.008
MaR1 (Attack vs. Interictal)	339.20 (273.26–593.32)	417.03 (358.56–745.10)	—	0.001

Data are presented as median (interquartile range, IQR). Statistical comparisons were performed using non-parametric tests (Mann–Whitney U test or Wilcoxon signed-rank test, as appropriate). IQR, interquartile range; RvD1, resolvin D1; MaR1, maresin 1. All *p* values < 0.05 indicate statistically significant differences between groups.

**Table 3:** Diagnostic Performance of Serum Resolvin D1 and Maresin 1 in Predicting Migraine Status.

Phase	Biomarker	Cut-off Value	Sensitivity % (95% CI)	Specificity % (95% CI)	PPV (95% CI)	NPV (95% CI)	AUC (95% CI)	*p*-Value
Attack Period	RvD1	≤8.51 ng/mL	78.2 (65.0–88.2)	66.0 (51.7–78.5)	70.5 (56.8–83.3)	74.5 (60.2–84.5)	0.719 (0.624–0.801)	<0.001
Attack Period	MaR1	≤460.02 pg/mL	72.7 (59.0–83.9)	71.7 (57.7–83.2)	72.7 (58.9–83.9)	71.7 (57.8–83.2)	0.730 (0.636–0.811)	<0.001
Interictal Period	RvD1	≤7.26 ng/mL	65.5 (51.4–77.8)	71.7 (57.7–83.2)	70.6 (56.3–81.6)	66.7 (52.8–79.6)	0.662 (0.550–0.750)	0.004
Interictal Period	MaR1	≤564.91 pg/mL	70.9 (57.1–82.4)	67.9 (53.7–80.1)	69.6 (55.7–81.5)	69.2 (55.1–81.0)	0.648 (0.631–0.737)	0.009

PPV: Positive predictive value; NPV: Negative predictive value; AUC: Area under the receiver operating characteristic curve; CI: Confidence interval; RvD1: Resolvin D1; MaR1: Maresin 1. ROC analysis indicates significant discriminative capacity of both biomarkers for migraine diagnosis.

## Data Availability

The data that support the findings of this study are available from the corresponding author upon reasonable request. Due to privacy and ethical restrictions, the data are not publicly available because they contain potentially identifiable patient information. Raw ELISA datasets, ROC curve source data, and anonymized demographic tables can be provided by the corresponding author upon reasonable request.

## Abbreviations

The following abbreviations are used in this manuscript:

RvD1	Resolvin D1
MaR1	Maresin 1
ROC	Receiver Operating Characteristic
AUC	Area Under the Curve
PPV	Positive Predictive Value
NPV	Negative Predictive Value
CI	Confidence Interval
IQR	Interquartile Range
ELISA	Enzyme-Linked Immunosorbent Assay
PUFAs	Polyunsaturated Fatty Acids
BMI	Body Mass Index
SD	Standard Deviation
